# On the Road to Salt Liberation: The Effect of Including Oyster Mushrooms and Sylvinite on the Quality of Traditional Beef Patties

**DOI:** 10.3390/foods15061013

**Published:** 2026-03-13

**Authors:** Gaston Sepulveda-Truan, Johanan Espinosa-Ramírez, Viridiana Tejada-Ortigoza, Rommy Díaz, Nestor Sepúlveda, Leonardo Almonacid, Ailin Martínez, Erick Scheuermann, Ruben Domínguez-Valencia, John Quiñones

**Affiliations:** 1 Doctoral Program in Agri-Food Sciences and Environment, Universidad de La Frontera, Av. Francisco Salazar 1145, Temuco 4811230, Chile; g.sepulveda10@ufromail.cl; 2 Centro de Tecnología e Innovación en Calidad de la Carne (CTI-Carne), Temuco 4780000, Chile; rommy.diaz@ufrontera.cl (R.D.); nestor.sepulveda@ufrontera.cl (N.S.); a.martinez26@ufromail.cl (A.M.); 3 School of Engineering and Sciences, Tecnologico de Monterrey, Av. Eugenio Garza Sada 2501 Sur, Monterrey 64849, NL, Mexico; johanan_er@tec.mx (J.E.-R.); viri.tejada@tec.mx (V.T.-O.); 4 Facultad de Ciencias Agropecuarias y Medioambiente, Universidad de La Frontera, Av. Francisco Salazar 11 01145, Temuco 4811230, Chile; leonardo.almonacid@ufrontera.cl; 5 Center for Biodiversity and Ecological Sustainability (C-BEST), Faculty of Agricultural Sciences and Environment, Universidad de La Frontera, Temuco 4811230, Chile; 6 Doctoral Program in Science Major in Applied Cellular and Molecular Biology, Universidad de La Frontera, Av. Francisco Salazar 01145, Temuco 4811230, Chile; 7 Department of Chemical Engineering, Universidad de La Frontera, Avenida Francisco Salazar 01145, Temuco 4811230, Chile; ericks@ufrontera.cl; 8 Centro Tecnolóxico da Carne, Parque Tecnolóxico de Galicia, Avda. Galicia nº4, 32900 San Cibrao das Viñas, Ourense, Spain

**Keywords:** hamburgers, novel food, less sodium, minerals, oyster mushroom, sylvinite, sensory analysis

## Abstract

This study evaluated the technological and sensory effects of incorporating oyster mushroom (*Pleurotus ostreatus*) powder and sylvinite as strategies to reduce salt content in beef patties while maintaining product quality. A 4 × 4 full factorial design was implemented to develop sixteen distinct formulations, evaluating the interaction between four levels of mushroom powder (0, 3, 5, and 10% *w*/*w*) as a partial meat replacer and four levels of sylvinite (0, 0.5, 1, and 2% *w*/*w*) as a NaCl substitute. To establish a baseline for comparison, control samples were prepared without sylvinite, with a fixed concentration of 1% NaCl. Patties were produced with low-fat content (6%), formed into 100 g portions, and evaluated in raw and cooked states. Physicochemical analyses included color (CIE L*, a*, b*), cooking yield, shrinkage, and texture profile analysis, while sensory quality was assessed by an expert panel and complemented with consumer discriminative tests, specifically a triangle test. Multivariate analysis revealed that mushroom powder significantly influenced color parameters, increasing redness and yellowness, whereas sylvinite tended to reduce color intensity; however, their interaction mitigated these effects at intermediate inclusion levels. Mushroom incorporation improved cooking yield and reduced hardness, particularly at 3–5% inclusion, enhancing elasticity and cohesiveness. Sensory results indicated that formulations containing 3–5% mushroom powder and up to 2% sylvinite achieved high overall acceptability. Consumer tests confirmed that these formulations effectively modulated saltiness and texture perception. Overall, the combined use of oyster mushroom powder and sylvinite represents a viable approach for developing reduced-sodium beef patties with acceptable technological and sensory properties.

## 1. Introduction

Today, hamburgers are a central component of processed meat products and fast food, driven by urbanization, lifestyle changes, and the demand for convenience. In this context, the consumption of processed meat has increased globally, now representing more than 40% of total meat production [1,2]. In the United States, beef patties account for approximately 17% of all unprocessed red meat consumed [3], while in countries like Portugal, 78% of university students prefer beef-based menus when opting for fast food [4]. Despite their widespread popularity, hamburgers are traditionally characterized by high levels of fat and sodium, which are essential for microbiological stability, texture development, and overall sensory acceptability [4].

Dietary sodium intake is a major risk factor for hypertension and cardiovascular disease. However, the scientific community continues to debate the optimal levels of restriction. Messerli et al. [5] reported a positive correlation between sodium intake and life expectancy across 181 countries, suggesting a “J-shaped” relationship where both extreme deficiency and excessive intake may increase health risks. Within this framework, food engineering is shifting toward a functional paradigm, namely the reformulation of meat products to reduce sodium without compromising expected sensory attributes. This transition requires innovative strategies that address the loss of palatability and structural integrity associated with sodium reduction.

One of the most promising mineral-based solutions is the use of potassium chloride (KCl). KCl can be sourced from sylvinite, a naturally occurring mineral composed of sodium chloride (NaCl) and potassium chloride [6]. Recent reports from AECOSAN [7] and the EFSA [8] have validated the safety of salts derived from sylvinite as food additives, which can be found in commercial forms as sylvinite-derived KCl, but, more recently, directly as sylvinite. Substituting a portion of NaCl with sylvinite-derived KCl can reduce sodium content to levels of 0.7–0.9% in grilled patties [9]. Although KCl can introduce a bitter aftertaste, research shows that this can be effectively masked through the synergistic effect of spices and onion, maintaining a high level of consumer acceptance [9].

Parallel to mineral substitution, the inclusion of edible mushrooms as biological meat extenders has gained significant attention. Mushrooms like *Agaricus* *bisporus* and *Pleurotus ostreatus* (oyster mushrooms) are rich in natural glutamates, which enhance the umami perception—the “fifth taste”—thereby compensating for the reduced saltiness [10,11]. Compared to traditional extenders like textured soy protein (TSP), mushrooms better preserve flavor harmony, moisture retention, and a “meat-like” texture [10]. Furthermore, incorporating mushrooms at levels of 25–45% has been shown to contribute to reducing energy density and increasing dietary fiber and bioactive compounds [12,13].

The synergy between mineral substitution (sylvinite) and biological extension (oyster mushrooms) represents a robust framework for the development of clean-label and healthier beef patties. The scientific literature contains few reports on the incorporation of sylvinite into meat products, and even fewer studies have evaluated its use in combination with oyster mushrooms. Therefore, the aim of the present study was to assess the effect of different concentrations of oyster mushroom powder, combined with sylvinite, on the physicochemical quality, sensory profile, and consumer perception of traditional beef patties.

## 2. Materials and Methods

### 2.1. Mushroom Powder Preparation

The mushrooms were obtained from local producers from Valdivia, Chile. The mushrooms were received and visually inspected (e.g., discoloration, physical damage, signs of insect damage), then cut into small pieces and dried at 45 °C for 48 h in a convection oven (Memmert UFP 800, Schwabach, Germany). The dried mushrooms were ground using a domestic grinder (Moulinex, Model A320R1, Ecully, France) to obtain a fine powder. The powder was estimated to have a particle size of 0.5 mm, as a 0.5 mm sieve was used to obtain a homogenous particle size. The moisture content was 4%, measured with a thermobalance (Mettler Toledo, Columbus, OH, USA, HE53). The mushroom powder was stored in vacuum-sealed bags at −20 °C until further use.

### 2.2. Burger Preparation

The base of the burgers was prepared using beef and pork back fat purchased in local markets in Temuco, Chile. The meat was ground using a meat grinder (Moulinex, Model A320R1, France) and mixed with the other ingredients. The formulation of the burger patties included beef (64–75.5% *w*/*w*), pork back fat (6% *w*/*w*) and water as ice (18% *w*/*w*). This resulted in beef patties with added pork fat or beef-pork blended patties, depending on national regulations [14,15].

The formulations were made with different concentrations of sylvinite (replacing NaCl) and mushroom powder (replacing beef), as follows: sylvinite (0, 0.5, 1, and 2% *w*/*w*) and mushroom powder (0, 3, 5 and 10% *w*/*w*). All treatments containing 0% sylvinite were formulated with 1% NaCl to serve as positive controls. The sylvinite was purchased in local markets in Temuco, Chile, as an approved salt substitute for human consumption. This sylvinite contains 65% NaCl, 30% KCl and other minerals, as indicated by the commercial brand [16]. To estimate the added sodium content of each formulation, calculations were based on the NaCl contribution of both the pure NaCl salt and the sylvinite. The intrinsic sodium contribution of the oyster mushroom powder was considered negligible (<10 mg/100g dry weight) based on standard nutritional profiles [17,18,19]. The estimated added sodium for each treatment was as follows: control burgers were estimated to have ~393 mg (1% NaCl), 0.5% sylvinite approx. 127 mg, 1% sylvinite ~255 mg and 2% sylvinite ~510 mg. Notably, this calculation indicates that formulations containing 2% sylvinite result in a higher total sodium content (~510 mg/100 g) than the 1% NaCl control (~393 mg/100 g).

A full 4 × 4 factorial design was used, with mushroom powder inclusion (0, 3, 5, and 10% *w*/*w*) and sylvinite inclusion (0, 0.5, 1, and 2% *w*/*w*) as fixed factors, resulting in a total of 16 formulations. The summary of each formulation is shown in Table 1. After the addition of all the ingredients, the formulations were mixed for 10 min using a food mixer (Oster, Model FPSTSM2712-033, Boca Raton, FL, USA) at low speed to obtain a homogeneous batter. The dough was then shaped into 100 g patties using a burger press (Weston, Model 07-0701-W, Southern Pines, NC, USA), and 50 burgers per treatment were prepared in separate batches to prevent cross-contamination. The burgers were stored at −20 °C in vacuum-sealed bags until further analysis.

Analyses were performed in raw and cooked patties; all the patties were stored for less than a week, and for every test, they were defrosted at 4 °C overnight. The cooking was performed in a preheated electric grill (Oster, Model CKSTGRFM18-033, Boca Raton, FL, USA) at 200 °C until reaching an internal temperature of 70 °C, monitored using a digital multi-thermometer.

### 2.3. Color Evaluation

The color of the raw and cooked burgers was measured using a colorimeter (Minolta CR-400, Osaka, Japan) with six points measured on the surface of each burger using three replications per treatment. The color parameters were recorded in the CIE L* a* b system, where L indicates lightness (0 = black, 100 = white), a* the redness/greenness (+a* = red, −a* = green), and b* the yellowness/blueness (+b* = yellow, −b* = blue). Hue (H*) and Chroma (C*) values were calculated using the following equations [20]:

(1) $$Hue={tan}^{-1}\left(\frac{b^{*}}{a^{*}}\right)$$

(2) $$Chroma=\sqrt{(a^{*}{)}^{2}+(b^{*}{)}^{2}}$$

### 2.4. Cooking Performance

Cooking loss (CL) and shrinkage (SH) of the burgers were determined by weighing and measuring the diameter of the raw and cooked burgers. The CL was calculated using the following equation obtained according to [21]:

(3) $$CL\left(\%\right)=\frac{W_{raw}-W_{cooked}}{W_{raw}}\times 100$$

(4) $$SH(\%)=\frac{D_{raw}-D_{cooked}}{D_{raw}}\times 100$$

To measure the change in diameter before and after cooking, every burger was photographed and measured digitally using ImageJ 2 2.3.0 software [22].

### 2.5. Texture Analysis

The texture analysis was performed on cooked burgers cooled to 25 °C using a CT3 Texture Analyzer (Brookfield Engineering, Middleboro, MA, USA). To measure texture, two analyses were performed: texture profile analysis (TPA) and Warner Bratzler shear force (WB). Automatic surface detection was used to set the height of the sample and the initial height of each test.

For WB, the samples were cut into two pieces (2.5 cm × 5.0 cm) per burger, and the samples were positioned perpendicular to the blade. Three burgers per treatment were used in this analysis. The probe used was a Warner Bratzler blade set (TA-SBA, Brookfield Engineering) with a test velocity of 4 mm/s, pre-test velocity of 2mm/s and trigger force of 1 N [23]. The recorded measurement was shear force (N).

For TPA, the samples were cut into four cylindrical samples (2.5 cm diameter) per burger, and four burgers per treatment were analyzed. The probe used was a 3.5 cm diameter cylinder (TA25/1000, Brookfield Engineering) with a test velocity of 3 mm/s for two cycles, a pre-test velocity of 2mm/s and 1 mm/s, compression to 75% of the original height, a trigger force of 1 N, and a 5 s recovery time [23]. The calculated measurements were hardness (N), springiness, cohesiveness, and chewiness (mJ) [24].

### 2.6. pH

The pH was measured using a digital pH meter (Hanna Instruments Inc., Cluj, Romania) previously calibrated with automatic temperature compensation and a glass penetration electrode adapted for meat and meat product measurements. Six points were measured per sample, with 3 replications per treatment.

### 2.7. Sensory Analysis

#### 2.7.1. Sensory Evaluation by Panel of Experts

A panel of 8 experts was recruited to analyze burger samples. The panelists were previously trained in the laboratory through prior training sessions and experimental trials. The burgers were cooked on an electric grill (Oster, Model CKSTGRFM18-033, Boca Raton, FL, USA) at 200 °C until reaching an internal temperature of 70 °C. The grilled samples were then cut into 4 cm × 4 cm squares and kept warm in plastic bags over water vapor using a thermostatic water bath (Bozhen, Shanghai, China, HH-6) at 80 °C until consumption. Each expert had mineral water and plain crackers to reduce carryover effects between samples. Each expert evaluated a total of nine samples in one session using an optimal balanced design created using the SensoMineR 1.28 package [25]. Randomization of the samples was performed with the same package, and each sample was presented with a random code to the evaluators.

The evaluation consisted of a Likert scale from 0 to 10, evaluating taste, texture, juiciness, flavor and global acceptance.

#### 2.7.2. Discrimination Evaluation by Consumers

From the sensory evaluation by the panel of experts, the five best-scoring treatments were chosen to be evaluated by consumers. To evaluate the effect of sylvinite and mushroom powder, two Directional Difference Tests were performed; in this test, each consumer answered a question regarding three samples (two from the same treatment group and one from another treatment), and the three samples were offered simultaneously. To orient participants in the test, two questions were used: (1) Which product of the three is saltier?, and (2) Which product of the three is softer in texture?

The burgers were cooked on an electric grill (Oster, Model CKSTGRFM18-033, Boca Raton, FL, USA) at 200 °C until reaching an internal temperature of 70 °C; the grilled samples were then cut into 4 × 4 cm squares and served immediately for consumption. The samples were presented coded (A, B and C) under blind conditions for the consumer.

The sample size was calculated considering a 40% probability of discrimination and a test power of 80%, resulting in a required sample size of 23. This calculation was made using the sensR 1.5 package [26]. As this test was performed with five treatments, 10 unique pairs were formed, which required a total of at least six comparisons per consumer. To minimize sensory fatigue, consumers were limited to three evaluations per session in a Balanced Incomplete Block Design [27], where the randomization of the consumers as well as the randomization of the pairs was done using the SensomineR 1.28 package [25]. This required a total experimental load of 69 evaluations, necessitating a panel size of *n* = 69 (23 × 3).

A total of 72 consumers completed the test in four sessions. The test was performed in an open environment, inviting people to public events. To participate, the consumers were screened to ensure they consumed beef patties at least once a week. The demographic description of the sample can be found in Table 2.

### 2.8. Statistical Analysis

All analyses and the previous estimation of the experimental design were performed using R software version 4.2.2 [28].

The data obtained from the different physical and chemical analyses were analyzed using a Permutational Multivariate Analysis of Variance (PERMANOVA), with mushroom and sylvinite inclusion levels as fixed factors, including their interaction, based on Euclidean distance, 999 permutations and sequential sums of squares via the vegan package [29]. When significant differences (*p* < 0.05) were found, pairwise PERMANOVA was used to compare the means, adjusted by the Benjamini–Hochberg method using the pairwiseAdonis package [30].

When the interaction between the inclusion of oyster mushroom and sylvinite was not significant in the PERMANOVA, the univariate effects on specific parameters were evaluated per factor, and the data were subsequently analyzed using Kruskal–Wallis analysis with the rstatix package [31]. When significant differences were found, post hoc multiple comparison analysis was conducted to compare between groups of the same factor (oyster mushroom inclusion or sylvinite inclusion) using the pgirmess package [32].

For the sensory evaluation (panel of experts), panel performance and Multivariate Analysis of Variance (MANOVA) with Holm’s adjustment for multiple comparisons were analyzed using the SensomineR 1.28 package [25]. To group the best treatments, a hierarchical cluster was used, and the analysis was performed using the Factoshiny 2.7 package [33].

For the discrimination analysis (consumers), consumer response performance and panel performance were analyzed using the sensR 1.5 package [26]. The data were later analyzed using a Bradley–Terry model to evaluate the effect of each inclusion level on consumer preference; when interaction effects were significant, the BradleyTerry2 package was utilized [34].

## 3. Results

### 3.1. Color Evaluation

The results of the individual measures of each treatment were analyzed by PERMANOVA analysis, and the results are shown in Table 3 (Table A1 and Table A2 show photographs of the burgers). The results indicate that the inclusion of oyster mushroom powder and sylvinite significantly affected the color parameters (a^*^, b^*^, L^*^) of the burgers. Furthermore, the interaction between the addition of the mushroom and the sylvinite was significant, as shown in Table 3.

To understand the interaction between the sylvinite and the oyster mushroom powder on the color parameters, a pairwise PERMANOVA was performed (Table 4).

The inclusion of the oyster mushroom powder demonstrated a significant effect on all the color parameters, with higher inclusion levels (5% and 10%) showing the most pronounced changes in redness (a*) compared with control samples (0%). Similarly, yellowness (b*) was significantly affected by the inclusion of the mushroom powder, with higher levels of inclusion leading to increased yellowness (ranging from 16.02 to 20.35).

No significant differences were observed in pH values across the different treatments, suggesting that the inclusion of sylvinite and mushroom powder did not substantially alter the pH of the burgers.

### 3.2. Cooking Performance

In cooking performance, no interaction effect was observed between sylvinite and oyster mushroom powder on cooking loss and shrinkage, as shown in Table 5.

Univariate analysis showed that the inclusion of 10% oyster mushroom powder significantly affected cooking loss compared with the control (0%). No significant differences were observed in shrinkage among the different levels of mushroom powder inclusion (Table 6).

For sylvinite inclusion, cooking performance was similar to that of the samples with oyster mushroom powder inclusion. The higher inclusion of sylvinite (2%) showed the lowest loss in cooking weight compared to the control. Similarly, no significant differences were observed in shrinkage values (Table 7).

### 3.3. Texture Analysis

No interaction effect was observed between sylvinite and oyster mushroom powder on the texture parameters measured (hardness, springiness, cohesiveness, chewiness and Warner Bratzler shear force).

Control samples (0% mushroom powder) showed the highest TPA hardness values (272 N), indicating greater resistance compared to samples with mushroom powder inclusion. A significant decrease in hardness was also observed in the Warner Bratzler shear force, where the control samples exhibited the highest values (Table 8).

Cohesiveness values were significantly affected by the 3% and 5% inclusion of the mushroom powder; nevertheless, chewiness values did not significantly increase at the 3% inclusion, which may indicate that this inclusion improves the internal structure of the burger.

### 3.4. Sensory Analysis

#### 3.4.1. Sensory Evaluation by Panel of Experts

**Figure 1:**
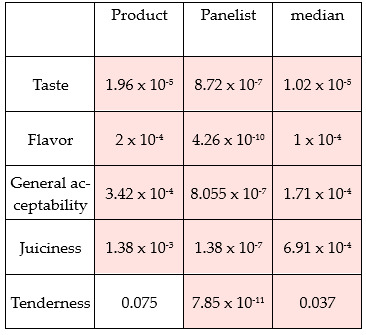
Panel performance (sorted by product *p*-value). Significant differences (*p* < 0.05) of MANOVA comparison are highlighted in pink.

Figure 1 summarizes the performance of the panel of experts. The panel of experts could significantly discriminate between all treatments (*p* < 0.05); however, they could not differentiate between the products in terms of tenderness (*p* = 0.75). This could be explained by the panel having more experience with whole meat than with burgers.

**Figure 2:**
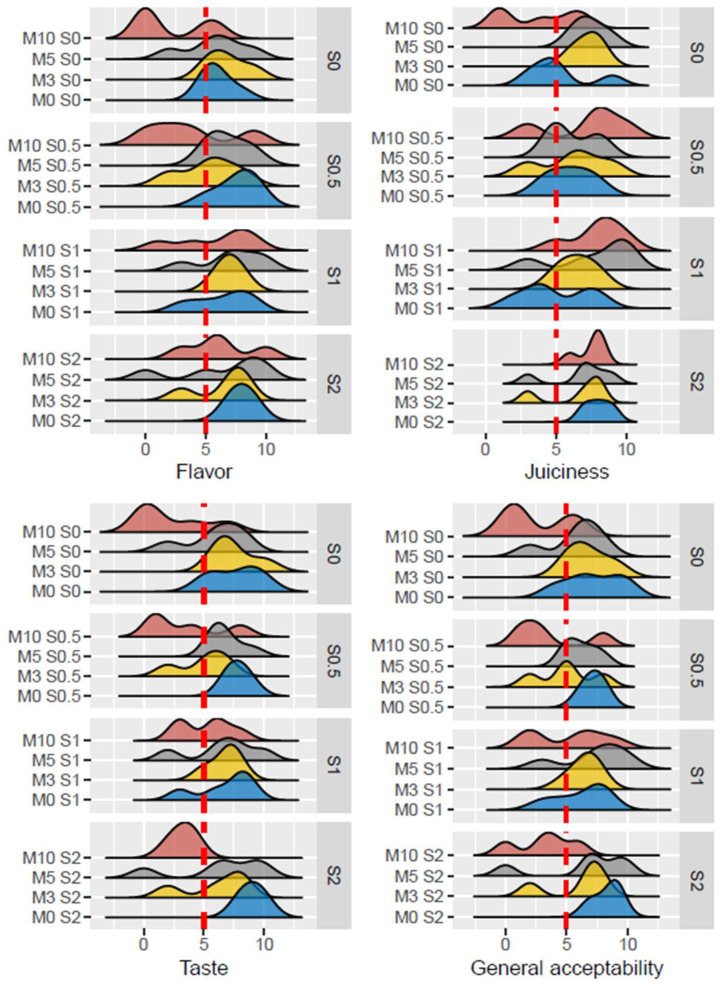
Distribution curves of sensory scores of burgers with different inclusion levels of oyster mushroom powder (0% (M0), 3% (M3), 5% (M5) and 10% (M10)) and sylvinite (0% (S0), 0.5% (S0.5), 1% (S1) and 2% (S2)) for the attributes of flavor, juiciness, taste and general acceptability.

Regarding the sensory properties of the products, all treatments containing 10% oyster mushroom powder showed the lowest scores for all evaluated parameters (taste, texture, juiciness, flavor and global acceptability) (Figure 2). The highest taste scores were obtained for two treatments: burgers with 0% mushroom powder and 2% sylvinite (M0S2 = 8.278 (adjusted mean)), and burgers with 0% mushroom powder and 0% sylvinite (M0S0 = 8.605 (adjusted mean)). The best flavor score was obtained by the treatment with 3% mushroom powder and 1% sylvinite (M3S1 = 8.194 (adj. mean)). The highest juiciness score was obtained for the treatment with 5% mushroom powder and 2% sylvinite (M5S2 = 8.111 (adj. mean)). The highest texture score was obtained for the treatment with 0% mushroom powder and 0% sylvinite (M0S0 = 8.430 (adj. mean)). Finally, the best score in global acceptance was obtained by the treatment with 0% mushroom powder and 2% sylvinite (M0S2 = 8.333 (adj. mean)).

**Figure 3:**
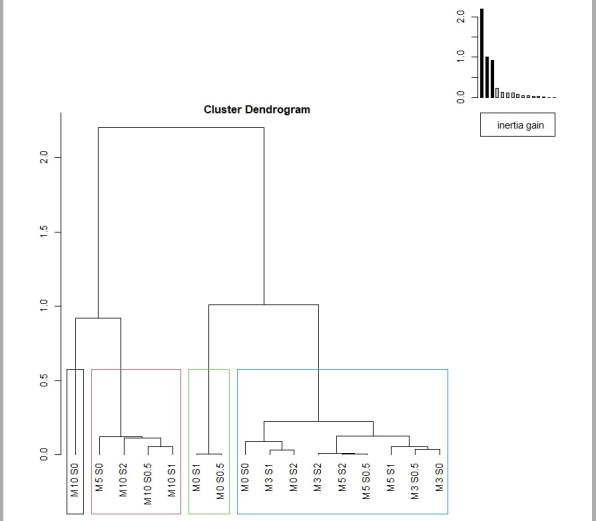
Hierarchical cluster analysis of the sensory evaluation of burgers with different inclusion levels of oyster mushroom powder (0% (M0), 3% (M3), 5% (M5) and 10% (M10)) and sylvinite (0% (S0), 0.5% (S0.5), 1% (S1), and 2% (S2)) for the attributes of flavor, juiciness, taste and general acceptability.

Results were subsequently analyzed using a hierarchical cluster analysis to group the best treatments (Figure 3). Four clusters were identified. The first cluster was designated “less acceptable”, and was characterized by those samples with the lowest scores in all evaluated parameters, with the treatment with 10% mushroom powder inclusion and 0% sylvinite (M10S0) being the most representative. The second cluster was called “less acceptable, but high in juiciness”, characterized by samples with low scores for all the evaluated parameters, but with high scores for juiciness, where the most representative treatments were those remaining with 10% oyster mushroom inclusion (M10S0.5, M10S1 and M10S2). The third cluster was designated “unattractive, dry and hard”, characterized by samples with low scores in global acceptance, flavor and taste; representative samples were those with no inclusion of oyster mushroom and 0.5% or 1% of sylvinite (M0S0.5 and M0S1). Finally, the fourth cluster was designated “highly acceptable”, characterized by samples with high scores for all the evaluated parameters, with the most representative treatments those with 0%, 3% and 5% of oyster mushroom powder inclusion with different levels of sylvinite inclusions (M0S0, M0S2, M3S0, M3S0.5, M3S1, M3S2, M5S0.5, M5S1 and M5S2).

#### 3.4.2. Discrimination Analysis by Consumers

From the clusters obtained in the sensory evaluation by the panel of experts, the treatments selected for further discrimination analysis by consumers were those with the highest barycenter value in cluster 4: M3S2 (0.67), M5S0.5 (0.75), M5S2 (0.91), M3S0.5 (0.93) and M3S1 (0.96).

All these treatments were tested for significant differences between them using a Hotelling T2 test. The results showed there were no differences between treatments M3S2 and M5S2 (*p* = 0.9537), nor between treatments M3S0.5 and M5S0.5 (*p* = 0.4738). Therefore, the final treatments selected for the discrimination analysis by consumers were M0S0, M3S1, M5S0, M5S0.5 and M5S2, with treatment M3S2 replaced by M0S0 (control sample) and treatment M3S0.5 replaced by M5S0.5 to have a better comparison.

To evaluate the performance of consumers, a chance-corrected beta-binomial model was used. The model revealed that subtle sensory differences were detected by consumers (*p* = 0.001). The sensory distance was 0.29, with an estimated 12.8% of the panel acting as true discriminators (*p*_d_ = 0.128) for the salt and texture test.

**Figure 4:**
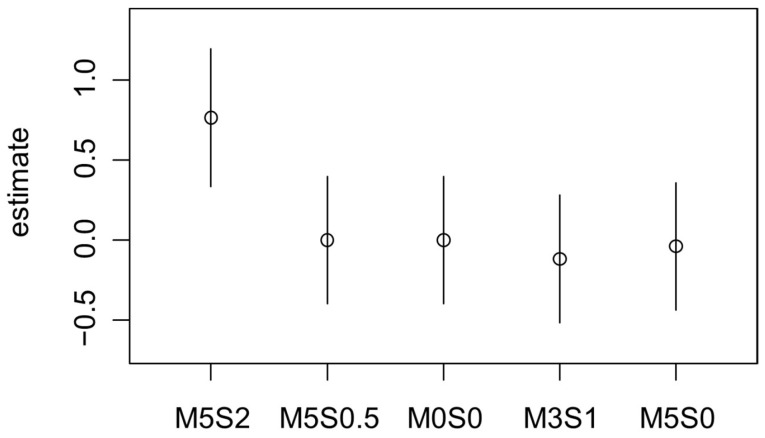
Quasi variances of the Bradley–Terry model of the discrimination analysis of burgers with 5% inclusion of oyster mushroom powder and 0% (M5S0), 0.5% (M5S0.5) and 2% (M5S2) sylvinite inclusion; 3% inclusion of oyster mushroom powder and 1% of sylvinite inclusion (M3S1) and control sample (M0S0) for the directional test in consumers about “Which product of the three is more saltier?”.

For the question “Which product of the three is saltier?”, a fitted Bradley–Terry model for pairwise comparison was used, with M0S0 (control sample) as a reference. The treatment with 5% oyster mushroom powder and 2% sylvinite was the most likely selected as saltier (B = 0.76, *p* < 0.01). In contrast, all the other treatments showed no significant differences from the reference sample (Figure 4). A second model was fitted to identify significant differences related to the inclusion of sylvinite in terms of preference. The model indicated that 2% sylvinite inclusion was the only level that differentiated the treatments from the control (*p* < 0.05).

**Figure 5:**
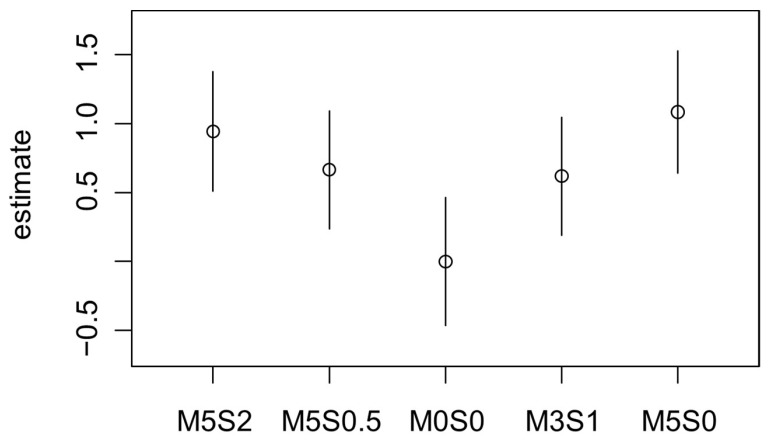
Quasi variances of the Bradley–Terry model of the discrimination analysis of burgers with 5% inclusion of oyster mushroom powder and 0% (M5S0), 0.5% (M5S0.5) and 2% (M5S2) sylvinite inclusion; 3% inclusion of oyster mushroom powder and 1% sylvinite inclusion (M3S1) and control sample (M0S0) for the directional test in consumers about “Which product of the three is softer in texture?”.

A third model was fitted to analyze the second question, “Which product of the three is softer in texture?” Samples M5S0 and M5S2 had the highest preference values (B = 0.83, *p* < 0.05 and B = 0.75, *p* < 0.05, respectively) (Figure 5). A fourth model was fitted to identify significant differences between the inclusion of oyster mushroom powder in the preferences for texture. The model indicated that 5% inclusion was the only level that differentiated treatment from control (*p* < 0.05).

## 4. Discussion

In this study, low-fat formulations (6%) were used, leading to a basal increase in redness (a*) and a reduction in yellowness (b*). This is explained by the high myoglobin content of lean meat, as well as by a reduction in the yellow color contributed by fat [35]. The inclusion of oyster mushrooms was shown to produce a significant increase in redness (a), yellowness (b), and lower lightness values (L). The addition of plant-based products can produce this effect due to the mushroom’s content of carotenoids [36,37], melanin pigments [38], polyphenol oxidase, and fiber [39]. These findings correspond with similar results regarding oyster mushroom replacements in chicken burgers [40,41], mortadella [42], and beef burgers [43,44], as well as the inclusion of other mushrooms in different meat products [39].

The addition of sylvinite alone did not result in significant color changes; similar results were reported in pork burgers [45] and beef meat emulsions with different rock salts [46], where no color differences were found between the use of different types of salt. In this study, there was only a significant change manifesting as a decrease in Chroma (C*) value at 2% inclusion in the treatment without oyster mushroom inclusion. This may be due to the potassium chloride content of the sylvinite, which has been shown to decrease color when used in meat matrices [47,48]; furthermore, sylvinite in its natural state can have a red to pink color, depending on the hematite content at the extraction site, and, therefore, depending on its purity, its effect on a* values may vary [49]. Nevertheless, in this study, a commercial brand was used, so the potential grade of impurity is expected to be low.

In this sense, the oyster mushroom was able to counteract the discoloration effect of the sylvinite and allowed for better C* values in the treatments, which is consistent with previous studies where the oyster mushroom achieved better color values than other mushrooms when interacting with salts [44].

The interaction between oyster mushroom inclusion and sylvinite caused treatments with different levels of sylvinite inclusion to exhibit reduced redness values and increased yellowness values. Likewise, the addition of sodium sources may not follow a linear pattern; variations in sodium concentration, as well as in protein structure, can result in effects at low concentrations that do not correspond with those at high concentrations [50].

The inclusion of mushrooms in meat products leads to a reduction in cooking loss [11,39,43,51,52]. This is mainly due to their capacity to retain water and fat within the product, attributed to their soluble dietary fiber content, as has been demonstrated with different plant ingredients [53,54,55,56]. However, some authors have reported that the inclusion of mushrooms in beef burgers can lead to an increase in cooking loss [57,58,59], especially when sodium content is reduced. This is because sodium induces a myosin extraction effect, which confers the capacity to contain water and fat within the meat product, generating the emulsion [60]. Vasquez Mejia et al. [46] tested different types of salt in pork burgers, finding that the significant effect was due to the inclusion level and not salt type.

Texture analysis indicated that the inclusion of oyster mushroom powder improved chewiness and springiness, and reduced hardness when included. This corresponds with other studies on beef burgers [12,44,57,61]. Regarding cohesiveness, the inclusion of 3% oyster mushroom increased values abruptly, which later decreased to reach a value similar to the control sample at the 10% concentration. Other authors have already reported that a similar or higher inclusion results in cohesiveness values similar to the control [43,57]. The increase in cohesiveness at lower inclusions (3% and 5%) may be due to the oyster mushroom’s ability to form filamentous structures, which is affected by the inclusion level, accompanying proteins, and mixing methodology, as well as the cooking process [62,63]. In this case, the low-fat content results in a higher proportion of proteins [55,64], so it is possible that this dense matrix, combined with an increase in fiber, generated a more compact structure [65]. Likewise, a lower oyster mushroom content in a high-protein matrix can limit protein oxidation, which affects texture [66]; increasing the oyster mushroom content results in higher polyphenol oxidase content, which can alter the protein matrix by oxidizing it, which would explain why in very dense matrices only the fiber exerts an effect [67]. Similarly, the presence of sodium chloride has been correlated with texture parameters in other studies [60,65,66]; however, in this study, there was no significant effect in this regard. Similarly, other studies have shown that the use of rock salts, compared to sodium chloride, does not significantly affect the texture of meat products [45,46]. Ruusunen et al. [64] indicate that sodium inclusion usually has a smaller effect on texture in low-fat contents and that fat inclusion has a greater effect in this aspect. Although instrumental analyses demonstrated significant differences between inclusion levels, the expert panel had difficulty differentiating tenderness among the products. In other studies, sensory perception of texture also demonstrated differences from instrumental results [57].

In general, the low ratings of the 10% oyster mushroom samples are consistent with findings in other studies: as mushroom inclusion increases beyond 5–8%, both flavor and texture cause rejection by evaluators [58]. In other mushroom species, this acceptance limit value may be even lower [39]. Additionally, *Pleurotus ostreatus* can be related to flavors that are considered foreign to meat products, which, in higher proportions, may cause rejection by consumers [11]. This is related to a neophobia effect and meat flavor expectation, where mushroom inclusion can predispose the consumer to reject flavors and odors foreign to meat [68,69], explained by a predisposition towards a traditional burger, which is often linked to a pleasurable feeling. While mushroom inclusion can be seen as an innovation, it will also generate a direct comparison, and possibly any change in flavor can generate rejection [70].

Regarding saltiness perception, consumers only showed greater interest in the sample with the highest sylvinite inclusion (2%), but not in the rest of the products. In other studies, the use of oyster mushrooms has been shown to effectively mask sodium reduction [57,71]; this is due to peptides found in the mushroom that generate a saltiness-enhancing effect [72]. Additionally, products with lower fat content tend to reduce saltiness perception [64], which could indicate that in products with higher fat inclusion, sylvinite could have a greater effect; in this sense, rock salts have demonstrated a greater saltiness perception effect in other products such as cheeses [73,74].

Rather than general optimization, future research must focus on the specific physicochemical dynamics in burgers. Specifically, microstructural analysis (e.g., SEM) is required to visualize the integration of the mushroom’s filamentous fibers within the beef myofibrillar network to confirm the mechanisms of textural improvement [75]. Additionally, because raw sylvinite contains trace minerals and oyster mushrooms possess highly active oxidative enzymes (such as polyphenol oxidase), future studies must evaluate the long-term lipid and protein oxidative stability of these formulations during refrigerated storage [43]. Finally, volatile profiling (GC-MS) of the beef–mushroom matrix is recommended to isolate the specific flavor compounds driving consumer rejection at higher (10%) inclusion levels [76]. Overall, this specific synergistic combination offers the meat industry a viable solution to meet the growing demand for reduced-sodium, low-fat beef products without compromising quality.

To the best of our knowledge, this could be the first scientific article of use of sylvinite in food. The research team noticed that, out of 284 mentions of “sylvinite” or “silvinite” in Scopus and 208 mentions in Web of Science, no research article described the use of sylvinite in food or meat. The only articles available are about mining.

## 5. Conclusions

The inclusion of oyster mushroom powder (3–5%) and sylvinite (at levels of 0.5–1%) provides a viable strategy for producing beef burgers with a reduced estimated sodium content, while maintaining acceptable sensory and physical properties. The oyster mushroom powder enhances color, texture, and cooking performance, while sylvinite effectively reduces sodium levels without compromising product quality. Sensory evaluations indicate that moderate inclusion levels (3–5%) are most likely selected by consumers, while higher levels (10%) may lead to rejection due to flavor alterations. Overall, this approach offers a promising avenue for developing healthier burgers and meat products that align with consumer preferences. The addition of the mushroom powder improved several quality parameters of the burgers, such as color, cooking performance, and texture, while the sylvinite enabled the reduction in the sodium content without negatively affecting the product’s quality or taste, as many other salt replacers do. The limited literature on rock salt or gourmet salt opens a door for future investigation. To the best of our knowledge, based on searches in Scopus and Web of Science (“sylvinite” or “silvinite”; February 2026), this is the first indexed report evaluating the use of food-grade sylvinite as a NaCl-replacing ingredient in beef patties (or meat products). Future research should focus on optimizing inclusion levels and exploring the synergistic effects of these ingredients to further enhance product quality and consumer acceptance, as well as to better understand the mechanisms behind the observed effects. Overall, this combination enables the development of healthier meat products, modulating saltiness and texture, providing a possible solution for the industry to meet the growing demand for reduced sodium and low-fat food products without compromising quality or consumer satisfaction.

## Figures and Tables

**Table 1:** Formulation of beef patties with pork fat with different concentrations of sylvinite and mushroom powder.

Ingredients	M0				M3				M5				M10			
	S0	S0.5	S1	S2	S0	S0.5	S1	S2	S0	S0.5	S1	S2	S0	S0.5	S1	S2
Beef Meat	75	75.5	75	73	72	72.5	72	70	70	70.5	70	69	65	65.5	65	64
Pork Back Fat	6	6	6	6	6	6	6	6	6	6	6	6	6	6	6	6
Water as Ice	18	18	18	18	18	18	18	18	18	18	18	18	18	18	18	18
Salt	1	0	0	0	1	0	0	0	1	0	0	0	1	0	0	0
Sylvinite	0	0.5	1	2	0	0.5	1	2	0	0.5	1	2	0	0.5	1	2
Mushroom Powder	0	0	0	0	3	3	3	3	5	5	5	5	10	10	10	10

M0: 0% mushroom powder; M3: 3% mushroom powder; M5: 5% mushroom powder; M10: 10% mushroom powder. S0: 0% sylvinite; S0.5: 0.5% sylvinite; S1: 1% sylvinite; S2: 2% sylvinite.

**Table 2:** Demographic characteristics of the consumers (*n* = 72).

Characteristic ^1^	*n* = 72
**Gen** **der**	
Male	51 (71%)
Female	19 (26%)
Non-binary	2 (3%)
**Age**	
<20	14 (19%)
21–30	33 (46%)
31–40	9 (13%)
>41	16 (22%)

^1^ Data are presented as *n* (%).

**Table 3:** Permutational Multivariate Analysis of Variance (PERMANOVA) of the inclusion of sylvinite (S), mushroom (M) and its interaction (*M* × *S*) on the color parameters (*L*∗, *a*∗, *b*∗) adjusted by the Benjamini and Hochberg method.

Source	Statistic	SES	Mean	PR (Perm)	Sig ^1^
Mushroom (M)	8.57	12.58	1.029	0.001	***
Sylvinite (S)	2.06	1.95	0.98	0.054	
Interaction (M × S)	2.29	3.77	1.01	0.002	**

^1^ Significance: * *p* < 0.05; ** *p* < 0.01; *** *p* < 0.001.

**Table 4:** Means (s’ Standard Error) of color parameters and pH influenced by oyster mushroom and sylvinite inclusion.

Mushroom ^1^ *a*∗		*b*∗	*L*∗	*C*∗	*h*∗	pH
0% Sylvinite (1% NaCl)						
M0	10.84 (0.96) ^a,b,c^	15.08 (0.48) ^a^	32.25 (0.94) ^a,b^	19.03 (0.62) ^a,b^	55.07 (2.48)	6.23 (0.13)
M3	11.66 (0.93) ^a,b,c^	17.46 (0.62) ^b,c^	37.69 (1.19) ^c^	21.12 (0.92) ^a,b,c^	56.88 (1.73)	5.99 (0.09)
M5	11.66 (0.93) ^a,b,c^	18.44 (0.44) ^c,d^	38.51 (1.65) ^c,d^	23.41 (0.54) ^c^	52.49 (2.84)	5.86 (0.12)
M10	12.94 (0.68) ^a,b^	18.39 (0.41) ^c^	35.22 (0.71) ^c,d,e^	22.68 (0.43) ^c,d^	55.03 (1.72)	6.11 (0.06)
0.5% Sylvinite						
M0	9.16 (0.49) ^c^	16.05 (0.46) ^a,b^	35.08 (1.65) ^a,b,c,d,e^	18.45 (0.49) ^b^	60.14 (1.50)	6.18 (0.07)
M3	12.35 (1.32) ^a,b,c^	16.58 (0.56) ^a,b,d,e^	35.07 (1.54) ^a,b,c,d,e^	21.30 (1.00) ^a,b,c^	54.67 (2.44)	6.04 (0.06)
M5	13.74 (1.11) ^a^	17.29 (0.42) ^b,c,f^	38.07 (1.65) ^a,c,e^	22.23 (0.84) ^c,e^	51.60 (2.27)	5.93 (0.13)
M10	13.34 (0.76) ^a,b^	17.92 (0.77) ^b,c,d^	35.24 (0.66) ^a,c,d,e^	23.12 (0.66) ^c,d^	54.47 (1.90)	6.12 (0.06)
1% Sylvinite						
M0	9.50 (0.63) ^c^	15.44 (0.50) ^a,d^	35.91 (0.96) ^e^	18.80 (0.83) ^a,b^	58.46 (1.94)	6.03 (0.09)
M3	11.25 (1.14) ^a,b,c^	14.86 (0.53) ^a^	30.76 (1.08) ^b^	18.88 (1.01) ^a,e,f^	54.21 (2.15)	6.02 (0.06)
M5	12.99 (0.94) ^b^	16.62 (1.29) ^a,b,c,d,e,f^	35.12 (1.79) ^a,b,c,d,e^	21.29 (1.28) ^a,b,c,d,e,f^	51.79 (2.60)	5.96 (0.12)
M10	12.27 (0.37) ^a,b^	18.49 (0.56) ^c,e^	34.14 (1.13) ^a,b,c,d,e^	22.36 (0.47) ^c,d^	55.89 (1.15)	6.10 (0.06)
2% Sylvinite						
M0	8.98 (0.39) ^c^	14.75 (0.84) ^a,f^	31.73 (1.27) ^a,b,d,e^	17.34 (0.82) ^f^	59.01 (1.24)	5.94 (0.10)
M3	10.84 (0.64) ^a,b,c^	16.77 (0.70) ^a,b,c,d,e,f^	36.76 (0.81) ^c^	20.16 (0.66) ^a,b,d,e^	56.95 (2.04)	5.98 (0.06)
M5	10.05 (0.33) ^c^	18.16 (0.89) ^b,c^	37.35 (1.59) ^a,c,d,e^	20.89 (0.76) ^a,c,e^	57.45 (4.23)	5.94 (0.10)
M10	12.97 (0.69) ^a,b^	18.61 (0.65) ^c,e^	35.22 (1.71) ^a,b,c,d,e^	22.79 (0.69) ^c,d^	55.16 (1.67)	5.94 (0.10)

Note: values represent Mean (Standard Error). ^1^ M0: 0% mushroom powder; M3: 3% mushroom powder; M5: 5% mushroom powder; M10: 10% mushroom powder. ^a,b,c,d,e,f^ Different letters in the same column represent significant differences (*p* < 0.05, adjusted by Benjamini and Hochberg method) between levels by PERMANOVA pairwise analysis.

**Table 5:** Permutational Multivariate Analysis of Variance (PERMANOVA) of the inclusion of sylvinite (S), mushroom (M) and its interaction (*M* × *S*) on the cooking performance.

Source	Statistic	SES	Mean	PR (Perm)	Sig ^1^
Mushroom (M)	4.56	3.55	1.08	0.014	*
Sylvinite (S)	0.51	2.65	1.08	0.026	*
Interaction (M × S)	1.33	0.53	1.02	0.242	

^1^ Significance: * *p* < 0.05; ** *p* < 0.01; *** *p* < 0.001.

**Table 6:** Change in the raw weight (g), cook weight (g) and cooking performance of the inclusion mushroom (M) at different levels of inclusion (0%, 3%, 5% and 10%).

Characteristic ^1^	M0	M3	M5	M10	*p*-Value
Raw weight (g)	96.93 (0.95) ^a^	100.13 (1.96) ^a,b^	100.00 (0.47) ^a,b^	101.60 (0.81) ^b^	0.002
Cooked weight (g)	68 (2) ^a^	75 (2) ^a,b^	81 (2) ^b^	82 (3) ^b^	0.002
Cooking loss (%)	29 (2) ^a^	25 (3) ^a,b^	19 (2) ^a,b^	20 (3) ^b^	0.021
Area lost (%)	28 (4)	27 (3)	24 (3)	31 (4)	0.5
Shrinkage (%)	14 (3)	13 (2)	13 (2)	17 (2)	0.5

Note: Means sharing the same letter within a row are not significantly different (Kruskal pairwise analysis, *p* > 0.05). Values represent Mean (Standard Error). ^1^ M0: 0% mushroom powder; M3: 3% mushroom powder; M5: 5% mushroom powder; M10: 10% mushroom powder.

**Table 7:** Change in the raw weight (g), cook weight (g), cooking loss, area lost, and shrinkage of the inclusion sylvinite (S) at different levels of inclusion (0%, 0.5%, 1% and 2%).

Characteristic ^1^	S0	S0.5	S1	S2	*p*-Value
Raw weight (g)	99.20 (0.75)	100.00 (0.83)	100.86 (2.06)	98.33 (1.37)	0.5
Cooked weight (g)	72 (3)	78 (3)	75 (2)	82 (3)	0.14
Cooking loss (%)	27 (3) ^a^	22 (2) ^a,b^	25 (2) ^a,b^	17 (2) ^b^	0.044
Area lost (%)	35 (4)	27 (3)	27 (3)	21 (4)	0.12
Shrinkage (%)	18 (3)	14 (2)	14 (2)	11 (2)	0.3

*Note:* Means sharing the same letter within a row are not significantly different (multi-comparison analysis, *p* > 0.05). Values represent Mean (Standard Error). ^1^ S0: 0% sylvinite; S0.5: 0.5% sylvinite; S1: 1% sylvinite; S2: 2% sylvinite.

**Table 8:** Texture profile analysis (TPA) and Warner Bratzel shear force analysis of cooked burgers with the inclusion of oyster mushroom powder at different levels of inclusion (0%, 3%, 5% and 10%).

Characteristics ^1^	M0	M3	M5	M10	*p*-Value
Cohesiveness	0.11 (0.05) ^a^	0.42 (0.02) ^b^	0.44 (0.03) ^b^	0.44 (0.02) ^b^	0.001
Chewiness (mj)	120 (58) ^a^	199 (38) ^b^	169 (55) ^a,b^	117 (30) ^a,b^	0.002
Springiness	0.16 (0.07) ^a^	0.62 (0.02) ^b^	0.64 (0.06) ^b^	0.61 (0.03) ^b^	0.004
Hardness (N)	272 (12) ^a^	94 (18) ^b^	80 (35) ^b^	54 (13) ^b^	<0.001
WB (N) ^2^	29 (9) ^a^	9 (1) ^b^	26 (10) ^a,b^	11 (1) ^b^	<0.001

*Note:* Means sharing the same letter within a row are not significantly different (multi-comparison analysis, *p* > 0.05). Values represent Mean (Standard Error). ^1^ M0: 0% mushroom powder; M3: 3% mushroom powder; M5: 5% mushroom powder; M10: 10% mushroom powder; ^2^ WB: Warner Bratzel shear force.

## Data Availability

The data presented in this study are available on request from the corresponding author due to privacy concerns.

## Appendix A

**Table A1:** Photos of raw samples of burgers with the inclusion of sylvinite (S) at different levels of inclusion (0%, 0.5%, 1% and 2%) and oyster mushroom (M) powder at different levels (0%, 3%, 5% and 10%).

	M0	M3	M5	M10
S0				
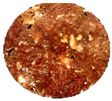
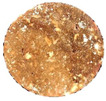
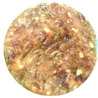
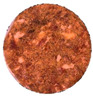
S0.5				
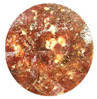
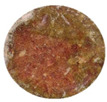
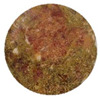
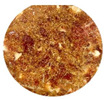
S1				
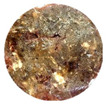
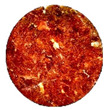
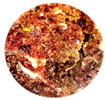
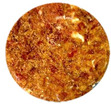
S2				
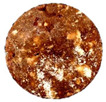
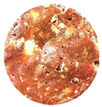
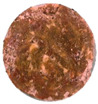
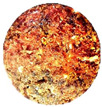

**Table A2:** Photos of cooked samples of burgers with the inclusion of sylvinite (S) at different levels of inclusion (0%, 0.5%, 1% and 2%) and oyster mushroom (M) powder at different levels (0%, 3%, 5% and 10%).

	M0	M3	M5	M10
S0				
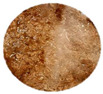
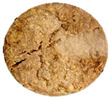
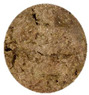
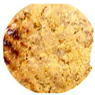
S0.5				
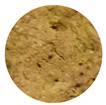
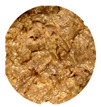
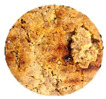
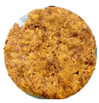
S1				
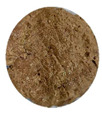
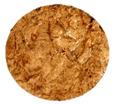
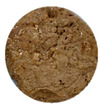
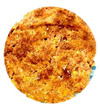
S2				
